# Shrimp Lipids Inhibit Migration, Epithelial–Mesenchymal Transition, and Cancer Stem Cells via Akt/mTOR/c-Myc Pathway Suppression

**DOI:** 10.3390/biomedicines12040722

**Published:** 2024-03-25

**Authors:** Chorpaka Thepthanee, Zin Zin Ei, Soottawat Benjakul, Hongbin Zou, Korrakod Petsri, Bhurichaya Innets, Pithi Chanvorachote

**Affiliations:** 1 Department of Food Science, School of Food Industry, King Mongkut’s Institute of Technology Ladkrabang, Bangkok 10520, Thailand; chorpaka.thep@gmail.com; 2 Center of Excellence in Cancer Cell and Molecular Biology, Faculty of Pharmaceutical Sciences, Chulalongkorn University, Bangkok 10330, Thailand; hushushin@gmail.com (Z.Z.E.); 6481004120@student.chula.ac.th (B.I.); 3 Department of Pharmacology and Physiology, Faculty of Pharmaceutical Sciences, Chulalongkorn University, Bangkok 10330, Thailand; 4 International Center of Excellence in Seafood Science and Innovation, Faculty of Agro-Industry, Prince of Songkhla University, Songkhla 90110, Thailand; soottawat.b@psu.ac.th; 5 College of Pharmaceutical Sciences, Zhejiang University, Hangzhou 310058, China; zouhb@zju.edu.cn; 6 Department of Pharmacology, Faculty of Medicine, Kasetsart University, Bangkok 10900, Thailand; korrakod.petsri@gmail.com

**Keywords:** lung cancer, shrimp lipids, migration, cancer stem cells, cholesterol, epithelial–mesenchymal transition (EMT)

## Abstract

Shrimp is a rich source of bioactive molecules that provide health benefits. However, the high cholesterol content in shrimp oil may pose a risk. We utilized the cholesterol elimination method to obtain cholesterol-free shrimp lipids (CLs) and investigated their anticancer potential, focusing on cancer stem cells (CSCs) and epithelial-to-mesenchymal transition (EMT). Our study focused on CSCs and EMT, as these factors are known to contribute to cancer metastasis. The results showed that treatment with CLs at doses ranging from 0 to 500 µg/mL significantly suppressed the cell migration ability of human lung cancer (H460 and H292) cells, indicating its potential to inhibit cancer metastasis. The CLs at such concentrations did not cause cytotoxicity to normal human keratinocytes. Additionally, CL treatment was found to significantly reduce the levels of Snail, Slug, and Vimentin, which are markers of EMT. Furthermore, we investigated the effect of CLs on CSC-like phenotypes and found that CLs could significantly suppress the formation of a three-dimensional (3D) tumor spheroid in lung cancer cells. Furthermore, CLs induced apoptosis in the CSC-rich population and significantly depleted the levels of CSC markers CD133, CD44, and Sox2. A mechanistic investigation demonstrated that exposing lung cancer cells to CLs downregulated the phosphorylation of Akt and mTOR, as well as c-Myc expression. Based on these findings, we believe that CLs may have beneficial effects on health as they potentially suppress EMT and CSCs, as well as the cancer-potentiating pathway of Akt/mTOR/c-Myc.

## 1. Introduction

Lung cancer has long been recognized as a malignant tumor with a high mortality rate, among which non-small cell lung cancer (NSCLC) accounts for approximately 85% of all lung cancer cases [1,2]. Although surgery combined with radiotherapy, chemotherapy, and targeted therapy has achieved certain results in treating NSCLC, the survival rate of advanced patients is relatively low [3,4]. The metastasis of lung cancer is one of the main causes of treatment failure and patient mortality [5]. Therefore, understanding the mechanism of cancer cell metastasis is extremely important.

The metastasis in many cancers is linked with the cellular process of epithelial–mesenchymal transition (EMT). This process involves the transformation of cells into mesenchymal motile cells and the loss of cell adhesion of the cells, which trigger a variety of pathogenic characteristics [5]. Several proteins are involved in the EMT process and contribute to cancer progression and metastasis, such as Snail, Slug, and Vimentin [6]. Transcription factors such as Snail and Slug are known to regulate the expression of EMT genes and play a crucial role in triggering the EMT process [6]. Overexpression of these transcription factors has been shown to be associated with poor prognosis and metastasis in many cancers, including lung cancer [7]. Vimentin is a mesenchymal marker that is upregulated during EMT and is associated with increased motility and invasiveness of cancer cells [5]. Recent studies have found that EMT is one factor that can induce cancer stem cell (CSC) characteristics. CSCs are among the most important factors contributing to cancer recurrence, metastasis, and drug resistance [8,9].

Many studies indicate that lung CSCs maintain their stemness, or stem cell-like properties, through their sustained expression of specific cell surface markers such as glycoproteins prominin-1 (CD133) and CD44-antigen (CD44), transcription factors like Sox2, and several signaling pathways [9,10]. These biomarkers are also associated with EMT and are involved in regulating the initiation and development of tumors [11]. The function of Sox2 involves regulating stem cell self-renewal and differentiation [12]. Furthermore, upregulation of Sox2 in a hypoxic environment can promote CD133 expression in lung cancer cells [13].

The upstream mechanism and the major controlling signaling pathway that determine stem cell properties in cancer have been extensively studied, especially as CSCs have emerged as key targets for novel anticancer therapeutics [11,14]. ATP-dependent tyrosine kinase, or protein kinase B (Akt), has been shown to regulate CSC properties in cancer [14]. The activation or augmentation of the active Akt signal has been shown to induce CSC phenotypes and promote the survival of the CSC population [15]. In addition, the stem cell transcription factors, including Oct4, Sox2, and Nanog, have been demonstrated to be downstream targets of the Akt signaling pathway [16]. The activation of the Akt signaling pathway promotes the formation of tumor-initiating cells by inducing EMT and facilitating cancer cell migration and invasion [17]. The suppression of the mammalian target of rapamycin (mTOR), the downstream target of Akt signaling, has been found to reduce invasion and cell migration as well as block CSCs from forming in NSCLC [18,19]. Moreover, c-Myc is a downstream target of the Akt/mTOR pathway, and its expression has been linked to both the EMT process and stem cell-like properties in cancer [20].

Shrimp byproducts, including the heads, shells, tails, and other parts of the shrimp that are not typically consumed as food, can make up to 50% of the shrimp’s total weight, and are often discarded as waste, raising environmental concerns [21]. A shrimp head contains oil or lipids, which are primarily composed of astaxanthin and various types of fatty acids, including polyunsaturated fatty acids (PUFAs) as well as cholesterol [22]. Astaxanthin has been widely investigated for its potential pharmacological properties such as antioxidant, antimicrobial, anticancer, anti-inflammatory, cardiovascular, antidiabetic, and skin-protective effects [23,24]. Essential fatty acids, including omega-3 PUFAs, are crucial for human health as they can help reduce the risk of heart disease and decrease inflammation [25]. Studies have reported that omega-3, particularly docosahexaenoic acid (DHA) and eicosapentaenoic acid (EPA), can inhibit cancer cell growth, induce cancer cell apoptosis, and exert an anti-angiogenic effect in several types of cancers [26,27,28]. Direct treatment with n-3 PUFAs can inhibit the growth of breast cancer cells and enhance differentiation [29]. The pro-differentiating effect of DHA was also confirmed in the human melanoma cell model [30]. However, epidemiological studies revealed that excessive cholesterol consumption raised the risk of cardiovascular disease and stroke [31,32] and cancer by encouraging cell proliferation, invasion, and metastasis in gastric, breast, and lung cancers [33,34,35]. Therefore, eliminating cholesterol can improve the quality of shrimp lipids. The purpose of this study was to investigate the effect of cholesterol-free shrimp lipids (CLs) on several key aspects of cancer progression, including cancer cell migration, EMT, and the expression of CSC-like phenotypes in NSCLC cell lines H460 and H292, as well as to identify the molecular mechanisms underlying these effects.

## 2. Materials and Methods

### 2.1. Preparation of Cholesterol-Free Shrimp Lipids (CLs)

#### 2.1.1. Shrimp Lipid Extraction and Fractionation Using Ethanol

The shrimp species used in this study was *Litopenaeus vannamei*, and it was obtained from Sea Wealth Frozen Food Co., Ltd. (Songkhla, Thailand), under frozen conditions (−18 °C). After thawing the shrimp heads, they were ground in a blender (National, Tokyo, Japan) to create a uniform paste. The method developed by Ei and coworkers [36] involved using a hexane/isopropanol mixture (1:1) to extract lipids from the shrimp paste. After extracting the lipids, the shrimp lipid extract was fractionated using ethanol in a separating funnel. The fractionation process involved adding ethanol to the lipid extract twice and separating the polar and non-polar lipid fractions. The lower layer of the non-polar lipid fraction was dissolved in 5 mL of hexane and kept at −20 °C, while the upper layer of the polar lipid fraction was evaporated using a rotary evaporator [37].

#### 2.1.2. Preparation of Silica Column

A glass column (diameter: 2 cm; height: 35 cm) was packed with dried silica gel (pore size: 7–230 mesh). Before packing, the bottom of the column was sealed using a pinch of cotton, which was then covered with a layer of celite (1 g) dispersed in 50 mL of hexane. Afterward, dried silica gel mixed with hexane was poured into the column and allowed to settle. Then, the column was purged with nitrogen gas to remove any air bubbles. Finally, the top of the silica gel was covered again with 1 g of celite. To prevent dryness and moisture absorption, the column was filled with hexane until it was used [37].

#### 2.1.3. Fractionation of Polar Lipids

**Figure 1:**
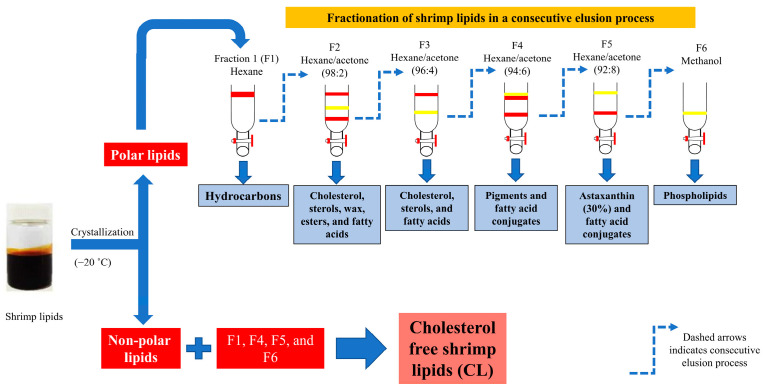
Preparation of cholesterol-free shrimp lipids (CLs) from the fractionation of shrimp lipids.

Different fractions of shrimp lipids were separated through the silica gel column using different solvents at several ratios [22]. Oil was first extracted from a shrimp cephalothorax [38]. Then, polar lipids were separated from the shrimp oil via ethanol crystallization at −20 °C. Thereafter, the obtained polar lipids (350 mg) were dissolved in the minimum volume of hexane, allowing them to settle at the top of the celite layer and then pass through the silica gel using hexane (100 mL), in which the first fraction (F1) was collected. To acquire the second, third, fourth, and fifth fractions, namely F2, F3, F4, and F5, respectively, the hexane/acetone mixture at varying ratios of 98:2, 96:4, 94:6, and 92:8 (*v*/*v*), respectively, was passed through the column. During loading, solvents were poured slowly around the wall of the column without disturbing the gel. Elution was accomplished when the reddish orange color in the eluent disappeared. The final fraction (F6) was eluted using methanol. The elution was performed at a constant flow rate of 4.5 mL/min. All the fractions were collected, and the elution volume for each fraction was recorded. Subsequently, evaporation was carried out using a rotary evaporator (Tokyo Rikakikai, Tokyo, Japan). The CLs were obtained by mixing non-polar lipids and fractions F1, F4, F5, and F6 (Figure 1). The mixed lipids were flushed with nitrogen, stored in an amber bottle, and tightly capped before being placed at −40 °C.

### 2.2. Fatty Acid Composition of CLs

The fatty acid composition of the sample was assessed using the Raju and Benjakul [22] technique. Initially, 10 mg of CLs was dissolved in 1 mL of hexane, followed by esterification through the addition of 200 µL of a 2 M methanolic sodium hydroxide solution at 50 °C for five min. After cooling, 200 µL of a 2 M methanolic hydrochloric acid solution was added. This mixture underwent 10 min of centrifugation at 3500× *g*, yielding a hexane phase for analysis. The hexane phase was introduced into an Agilent GC 7890B gas chromatograph (Agilent Technologies, Santa Clara, CA, USA) with an injection temperature of 250 °C. The chromatographic conditions included an initial column temperature of 80 °C, with a programmed 4 °C/min increase over 40 min to 220 °C, and then to 240 °C. The compounds were detected at a flame ionization detector temperature of 270 °C, and the peaks were identified using authentic standards. The obtained results were expressed in terms of grams per 100 g (g/100 g). Table 1 provides a comprehensive presentation of the fatty acid composition and content of the CLs extracted from the cephalothorax of Pacific white shrimp, including their respective quantitative values.

### 2.3. Preparation of CL Stock Solution

CL 100 mg/mL stock solutions were prepared by dissolving the CLs in dimethyl sulfoxide (DMSO) and storing them at −20 °C. To achieve the experimental concentrations (ranging from 0 to 500 μg/mL), the stock solution was diluted in a culture medium. DMSO content in the final solution was 0.2%.

### 2.4. Cell Cultures

Human NSCLC H460 and H292 cells and human keratinocyte HaCaT cells were incubated at 37 °C with 5% CO_2_, which provided a controlled environment for cell growth and survival. H460 and H292 were cultured in Roswell Park Memorial Institute (RPMI) 1640 medium (Gibco, Grand Island, NY, USA), whereas HaCaT cells were cultured in Dulbecco’s Modified Eagle Medium (DMEM) (Gibco, Grand Island, NY, USA). The RPMI and DMEM were supplemented with 2 mM of L-glutamine (Gibco, Grand Island, NY, USA) and 10% fetal bovine serum (FBS) (Merck KGaA, Darmstadt, Germany).

### 2.5. Cell Viability Assay

H460, H292, and HaCaT cells were seeded in 96-well plates at 1 × 10^4^ cells per well and allowed to adhere for 16 h at 37 °C. Cells were treated with various concentrations of CLs (0–500 µg/mL) at varying time periods of 24, 48, and 72 h. A total of 100 µL of MTT solution (0.4 mg/mL) was added to each well. The plates were then incubated for 3 h at 37 °C. The MTT solution was removed, and the formazan crystals were dissolved in 100 µL of DMSO. The intensity of the purple formazan was then assessed using a microplate reader at a wavelength of 570 nm.

### 2.6. Nuclear Staining Assay

For screening apoptotic cell death using double staining with Hoechst 33342 and propidium iodide (PI), H460, H292, and HaCaT cells (1 × 10^4^ cells/well) were seeded onto 96-well plates for 12–16 h. The cells were treated with various doses of CLs (0–500 µg/mL) for 24, 48, and 72 h. The cells were then labeled with Hoechst 33342 (10 g/mL) and PI (5 g/mL) for 30 min at 37 °C. The staining allowed for the visualization and imaging of apoptotic cells, which appeared bright blue due to the Hoechst 33342 staining, while necrotic cells appeared red due to the PI staining using fluorescence microscopy (Nikon ECLIPSE Ts2; Tokyo, Japan).

### 2.7. Wound-Healing Assay

NSCLC cells (3 × 10^4^ cells/well) were seeded onto 96-well plates for 24 h at 37 °C. A wound was produced by creating a gap in a straight line on the cell monolayer in each well using a 20 μL sterile plastic micropipette tip. The cells were then treated with various doses of CLs (0–500 µg/mL) for a period of up to 72 h. Images of the cells were taken at the specified time points of 0, 24, 48, and 72 h after treatment, and the wound space was measured using Image J software version 1.52a.

### 2.8. Migration Assay

A transwell migration assay was performed to test the migratory activity of the cells. The cells were treated for 48 h. Then, the cells were trypsinized and resuspended in serum-free media. The lower chamber of each transwell insert was filled with 600 µL of RPMI media containing 10% FBS, and the upper chamber of the insert was then filled with cells at a density of 1 × 10^5^ cells per well. After 16 h, the culture medium was removed from the transwell inserts, and the cells were washed twice with PBS to remove any residual media and serum. The cells were then fixed with 3.7% formaldehyde for 15 min to preserve their cellular structure and prevent any further migration. The cells were then permeabilized with 100% methanol for 15 min to allow for crystal violet staining to penetrate the cells and adhere to the cellular components. Next, the cells were stained with 0.5% crystal violet in 25% methanol for 10 min. The membranes were washed several times with PBS to remove any excess dye. The cells that remained in the upper chamber of the transwell insert were removed using cotton swabs. Finally, images of the cells that moved through the insert’s pores were captured using a 10× light inverted microscope.

### 2.9. Western Blot Analysis

Western blot analysis was used to detect and quantify specific proteins in a sample. NSCLC cells (H460 and H292) were plated at a density of 2 × 10^5^ cells per well in 6-well plates at 37 °C for 24 h and treated with various doses of CLs (0–500 µg/mL) for 48 h. The cells were washed with cold PBS and lysed using RIPA buffer containing protease inhibitors, Triton X, and PMSF for 40 min on ice. The total protein content of the cell lysate was quantified using a BCA protein assay kit (Pierce Biotechnology, Rockford, IL, USA). Equal amounts of protein sample were loaded onto SDS polyacrylamide gel and electrophoresed. The proteins were then transferred from the gel onto a polyvinylidene difluoride (PVDF) membrane using an electric field. The membrane was then blocked with 5% skim milk in TBST for 1 h to prevent non-specific binding of antibodies to the membrane. Next, primary antibodies, Vimentin (Cell Signaling, #5741, 1:1000), Snail (Cell Signaling, #3879, 1:1000), Slug (Cell Signaling, #9585, 1:1000), mTOR (Cell Signaling, #2983, 1:1000), p-mTOR ser2448 (Cell Signaling, #5536, 1:1000), Akt (Cell Signaling, #9272, 1:1000), p-Akt ser473 (Cell Signaling, #4060, 1:1000), CD44 (Cell Signaling, #3570, 1:1000), c-Myc (Cell Signaling, #18583, 1:1000), CD133 (Abcam, #ab19898, 1:1000), Sox2 (Cell Signaling, #3579, 1:1000), and β-actin (Cell Signaling, #4970, 1:1000), were incubated with the membrane overnight at 4 °C. The primary antibody bonded to the protein, and any unbound antibody was washed three times with TBST. A secondary antibody conjugated to an enzyme, or a fluorescent tag, was then added and incubated for 2 h at ambient temperature. Finally, protein bands were detected by adding a chemiluminescent substrate and were exposed to X-ray film. The intensity of the protein bands was quantified using Image J software to determine the level of protein expression in the sample.

### 2.10. Immunofluorescence Assay

Cells were seeded in a 96-well plate at a density of 5 × 10^3^ cells/well, and they were incubated overnight to allow them to adhere to the plate. After the cells were treated with CLs (0–500 µg/mL) for 48 h, they were washed with PBS and fixed with 4% paraformaldehyde in PBS for 15 min. Next, they were permeabilized with 0.5% Triton-X in PBS for 5 min and blocked with 10% FBS in PBS for 1 h. They were then probed with primary antibodies (Vimentin, Snail, Slug, mTOR, p-mTOR, Akt, p-Akt, CD44, c-Myc, CD133, and Sox2) overnight at 4 °C. After incubation with the secondary antibody and staining with Hoechst 33342 for 1 h at room temperature, the cells were rinsed with PBS, fixed with 4% paraformaldehyde in PBS, and mounted using 50% glycerol. The images were obtained using a fluorescence microscope, and Image J software was used to analyze the fluorescence intensity of stained cells.

### 2.11. Spheroid Formation Assay

Cells were pre-treated with CLs (0–500 µg/mL) for 48 h, and then they were plated at a density of 5 × 10^3^ cells per well in a 24-well ultra-low attachment plate. Primary tumor spheroids were captured after incubation for 3 and 7 days by a phase-contrast microscope (Nikon ECLIPSE Ts2; Tokyo, Japan). Then, spheroids were trypsinized and resuspended into a single cell. The 5 × 10^3^ cells/mL were seeded onto 24-well ultra-low attachment plates for 10 days to form secondary CSC-enriched spheroids and photographed at day 3, day 7, and day 10. The secondary spheroids were then transferred to a 96-well ultra-low attachment plate, with one spheroid per well, and treated with various concentrations of CLs. The changes in their size and shape were monitored at 0, 1, and 2 days using an inverted microscope. The single spheroids were stained with Hoechst 33342 and PI for 15 min on day 2, to assess their viability and proliferation, and imaged using a fluorescent microscope.

### 2.12. Statistical Analysis

The mean ± standard deviation (SD) of three independent results was reported. GraphPad Prism 5 (GraphPad Software, San Diego, CA, USA) was used to conduct an analysis of the statistical differences between the groups using ANOVA, followed by Dunnett’s multiple comparisons test for individual comparisons. A graph of all data was also generated. A *p*-value of less than 0.05 was considered statistically significant.

## 3. Results

### 3.1. Cytotoxicity Effect of CLs on H460 and H292 Lung Cancer Cells

**Figure 2:**
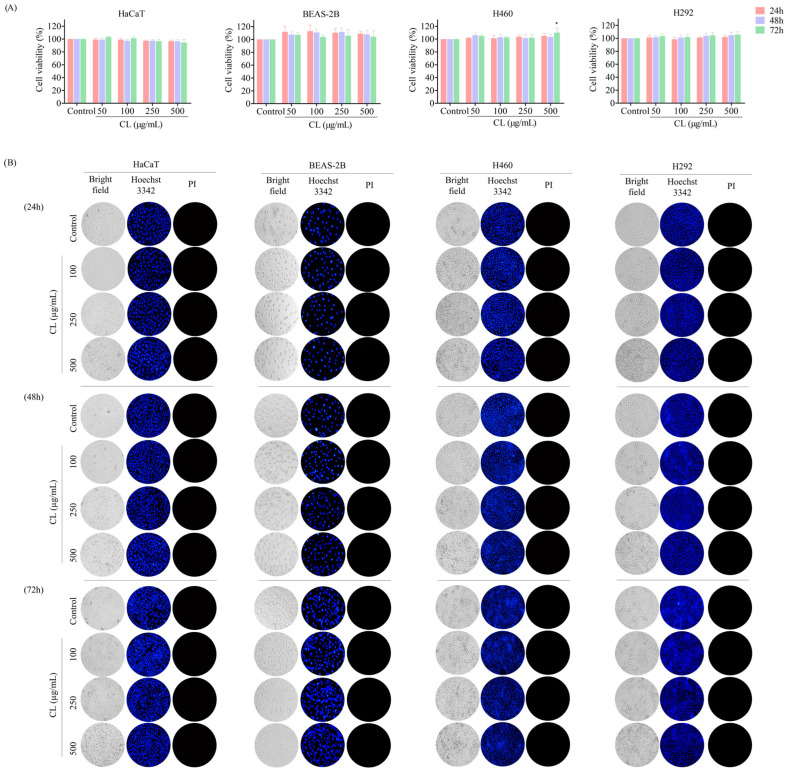
The cytotoxic effect of cholesterol-free shrimp lipids (CLs) on both a non-tumorigenic epithelial cell line from human bronchial epithelium cells (BEAS2B) and non-small cell lung cancer (NSCLC) cells using an MTT assay for cell viability and Hoechst 33342/PI staining for apoptotic and necrotic cell identification. (**A**) A total of 1 × 10^4^ of BEAS2B, H460, and H292 cells were seeded in 96-well plates and treated with various concentrations of CLs (0–500 µg/mL) for 24, 48, and 72 h. Cell viability was assessed by MTT assay. Percentages of cell viabilities were calculated based on the MTT assay results. (**B**) Apoptotic and necrotic cells were identified through co-staining with Hoechst 33342 and propidium iodide (PI). Images were identified by using a fluorescence microscope to visualize the stained cells. The data are presented as the means ± SD (*n* = 3). * *p* < 0.05, compared with the control group.

As the toxicity of a compound may interfere with the interpretation of results, the non-toxic concentrations of CLs to be used in the subsequent experiments were investigated. For comparison, the cytotoxic effect of CLs on normal cell lines (keratinocyte HaCaT cells) was determined. The cells were treated with CLs (0–500 μg/mL) for 24, 48, and 72 h. An MTT assay was employed to evaluate cell viability. The results revealed that the HaCaT cells were unaffected by CLs at concentrations of 0 to 500 μg/mL (Figure 2A). Moreover, the percentage of cell viability of the NSCLC cells (H460 and H292) did not significantly decrease in a dose- and time-dependent manner (Figure 2A).

Nuclear staining and fluorescence microscopy are commonly used techniques to visualize apoptotic cells and the changes that occur in the nucleus during the apoptotic process [39]. Double staining with Hoechst 33342 and propidium iodide (PI) is a commonly used method to screen for apoptotic cell death. Hoechst 33342 is a fluorescent dye that specifically binds to DNA, allowing for the visualization of nuclear morphology, while propidium iodide is a membrane-impermeable dye that stains only cells with damaged plasma membranes. In apoptotic cells, the nucleus typically undergoes condensation and fragmentation, which can be observed by labeling with Hoechst 33542, showing blue fluorescent light on condensed nuclei. In contrast, cells that have lost plasma membrane integrity, as in necrotic or late-stage apoptotic cells, will stain positive for PI. In addition, the nuclei of healthy cells are normally spherical, and their DNA is equally dispersed [40]. Hence, the presence of apoptotic and necrotic cells was assessed by Hoechst 33342/PI nuclear staining to further confirm CLs were not cytotoxic to normal and NSCLC cells at doses ranging from 0 to 500 μg/mL.

We treated normal and NSCLC cells with CLs at concentrations ranging from 0 to 500 μg/mL for 24, 48, and 72 h, and then stained with Hoechst 33342/PI. The result revealed that CLs had no effect on either apoptosis or necrosis-induced cell death in HaCaT, H460, and H292 cells (Figure 2B). The non-toxic doses of CL were then used in the following experiments.

### 3.2. CLs Inhibited Migration in H460 and H292 Lung Cancer Cells

**Figure 3:**
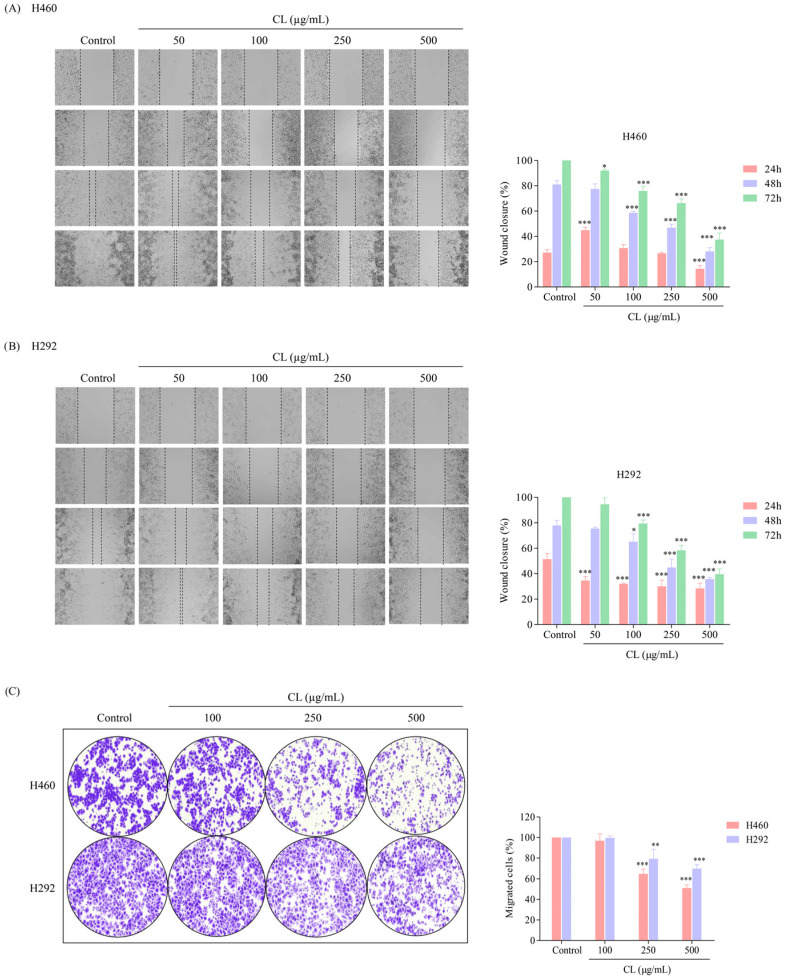
The migratory inhibitory effect of cholesterol-free shrimp lipids (CLs) on non-small cell lung cancer (NSCLC) cells using wound-healing and migration assays. Wound spaces were generated in (**A**) H460 and (**B**) H292 cells treated with various concentrations of CLs (0–500 µg/mL). Images were captured at 0, 24, 48, and 72 h using a microscope. The relative wound width was analyzed using Image J software version 1.52a. Histograms were generated to exhibit the wound closure rate, providing a visual representation of the inhibitory effect of CLs on H460 and H292 cell migration. (**C**) A migration assay was conducted to evaluate the migratory potential of pre-treated cells. H460 and H292 cells were seeded in upper chambers with RPMI-1640, containing 1% FBS, while the lower chambers were loaded with RPMI-1640 containing 10% FBS. After 16 h, migrated cells were stained, counted, and quantified using Image J software version 1.52a. Data are represented as the means ± SD (*n* = 3). * *p* < 0.05, ** *p* < 0.01, *** *p* < 0.001 when compared with the control group.

In order to evaluate the effect of CLs on migratory behavior, a wound-healing assay was conducted. Briefly, a confluent monolayer of NSCLC cancer cells was scratched to create a “wound”, and then the cells were treated with non-toxic concentrations of CLs (0–500 μg/mL) for 24, 48, and 72 h. In the case of H460 cells, cell migration was inhibited by CLs at the highest dose for 24 h (Figure 3A). However, CLs at concentrations of 50–500 μg/mL significantly decreased the migration of the H292 cells after 24 h (Figure 3B). Furthermore, the larger wound space detected in H460 and H292 cells incubated with 100–500 μg/mL CLs suggested an inhibitory effect on cell motility, while 50 g/mL CLs had no significant effect on cell migration for 48 and 72 h (Figure 3A,B). The results suggest that CLs have differential effects on cell migration depending on the cell type and concentration used.

In the next experiment, we tested the ability of lung cancer cells to migrate through a membrane filter in the presence of different gradients of FBS percentages in the RPMI medium. FBS is commonly used to promote cell migration by providing essential nutrients and growth factors [41]. The NSCLC cells were allowed to migrate for 16 h, and the non-motile cells on the upper part of the filter membrane were discharged. The migrated cells on the lower part of the membrane were stained with crystal violet. The results revealed that untreated H460 and H292 cells demonstrated a high migration rate and were capable of passing through the membrane filter (Figure 3C). However, when the cells were treated with CL, their ability to migrate was significantly decreased in a dose-dependent manner. This suppression of migration was observed when the cells were exposed to increased CL concentrations. These results suggest that CL might be an effective treatment for suppressing lung cancer cell migration and potentially preventing metastasis.

### 3.3. The Effect of CL Treatment on the EMT of NSCLC Cells

**Figure 4:**
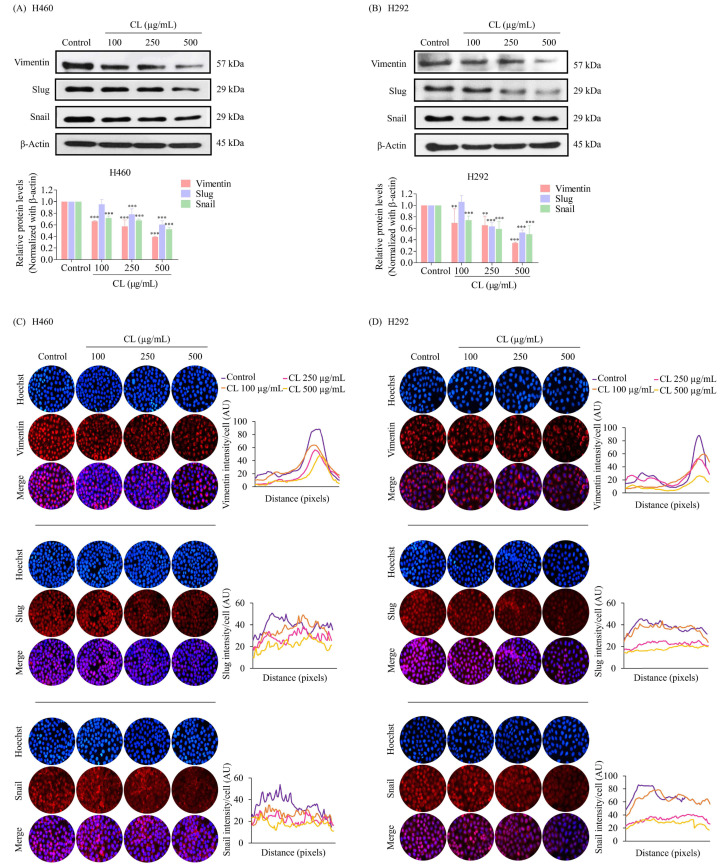
Cholesterol-free shrimp lipids (CLs) reduce the expression of the epithelial–mesenchymal transition (EMT) marker in non-small cell lung cancer (NSCLC) cells. (**A**) H460 and (**B**) H292 cells were treated with various concentrations of CLs (0–500 µg/mL) for 48 h. The expressions of key EMT markers, including Vimentin, Slug, and Snail, were assessed by Western blotting. β-actin was utilized as a loading control to ensure equal loading of the protein samples. Densitometry analysis was performed for each protein, and the results are presented as relative protein levels compared to untreated control cells. Uncropped blots can be found in Supplemental Figure S1. Immunofluorescence confirmation of decreased the expression of EMT markers (Vimentin, Slug, and Snail) in (**C**) H460 and (**D**) H292 cells treated with CLs. This analysis included the localization and expression patterns of Vimentin, Slug, and Snail. H460 and H292 cells were treated with various concentrations of CLs (0–500 µg/mL) for 48 h. Fluorescence imaging was conducted to visualize the cellular distributions of Vimentin, Slug, and Snail. The red signal represents the staining of corresponding proteins, while the blue signal indicates nuclear DNA staining using Hoechst 33342. The graph illustrates the quantitative analysis of the fluorescence intensities of Vimentin, Slug, and Snail in H460 and H292 cells. The data were captured using a fluorescence microscope and analyzed with Image J software version 1.52a. Data are represented as the means ± SD (*n* = 3). ** *p* < 0.01 and *** *p* < 0.001 compared with untreated cells.

EMT has been shown to facilitate the dissemination of cancer cells from the primary tumor, invade tissues, and spread to form secondary tumors [5]. To determine whether CLs affected tumor cell migration by blocking EMT, we detected the expression of the key EMT regulatory proteins in NSCLC cells. The cells were treated with CLs at concentrations ranging from 0 to 500 µg/mL for 48 h, and Western blotting was used to detect the protein expression levels of Snail, Slug, and Vimentin. The results showed that in both H460 and H292 cells, CL treatment significantly deregulated the expression of EMT transcription factors Snail and Slug compared to the control group (*p* < 0.05) (Figure 4A,B). Additionally, the expression of Vimentin, a mesenchymal marker, was also significantly decreased in both cell lines. These findings suggest that CLs may inhibit lung cancer cell migration by blocking EMT.

Immunofluorescence staining experiments further confirmed the treatment of CL-induced downregulation of EMT-associated markers. The results revealed that an intermediate filament called Vimentin, primarily found in the cytoplasm, was significantly reduced after H460 and H292 cells were treated with CLs (Figure 4C,D). We also discovered that Snail and Slug, important EMT-inducing transcription factors found in both the cytoplasm as well as in the nucleus, were clearly reduced by exposure to CLs (Figure 4C,D). These results provide further evidence that CLs could effectively inhibit EMT in NSCLC cells, potentially reducing their ability to migrate and spread.

### 3.4. CLs Suppress Cancer Stem Cells in NSCLC Cells

**Figure 5:**
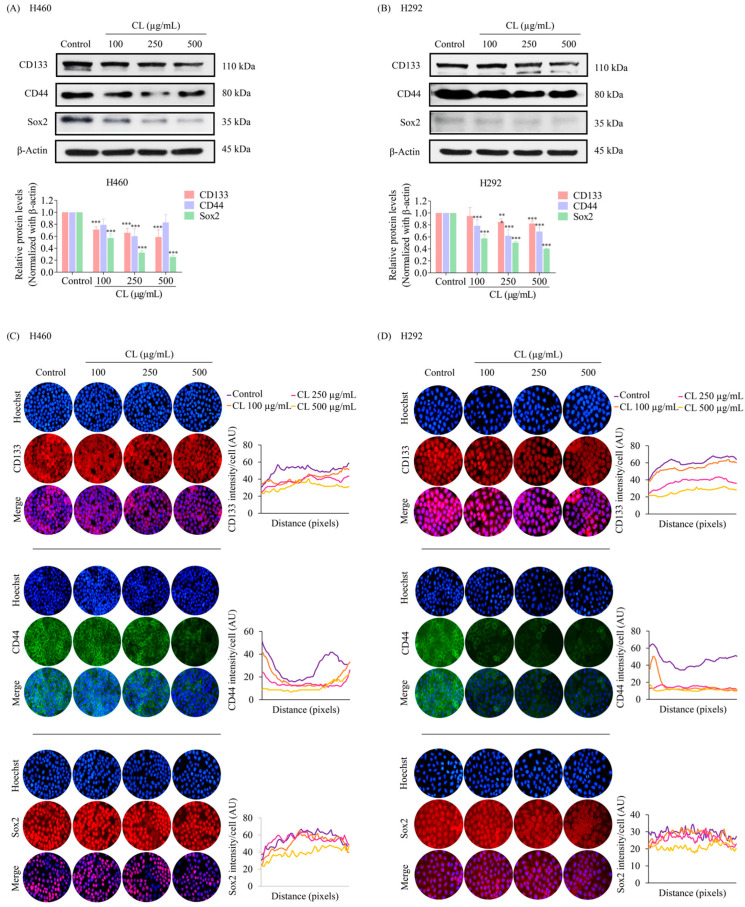
Cholesterol-free shrimp lipids (CLs) suppress cancer stem cells (CSCs) in non-small cell lung cancer (NSCLC) cells. (**A**) H460 and (**B**) H292 cells were treated with various concentrations of CLs (0–500 µg/mL) for 48 h. The expressions of CD133, CD44, and Sox2, were assessed by Western blotting. β-actin was utilized as a loading control to ensure equal loading of the protein samples. Densitometry analysis was performed for each protein, and the results are presented as relative protein levels compared to untreated control cells. Uncropped blots can be found in Supplemental Figure S2. To confirm the decreased expression of CSC markers (CD133, CD44, and Sox2) in (**C**) H460 and (**D**) H292 cells treated with CL, immunofluorescence staining was performed. Cellular distribution and expression patterns were visualized using a fluorescence microscope. Red signals represent the staining of CD133 and Sox2 proteins, green signals represent CD44 protein staining, and blue signals indicate nuclear DNA staining using Hoechst 33342. The graph illustrates the quantitative analysis of fluorescence intensity for CD133, CD44, and Sox2 in the H460 and H292 cells. The data were captured using a fluorescence microscope and analyzed with Image J software version 1.52a. Data are represented as the means ± SD (*n* = 3). ** *p* < 0.01 and *** *p* < 0.001 compared with untreated cells.

During EMT, cancer cells can acquire stem cell-like properties, including the ability to self-renew and differentiate, making them more invasive and resistant to therapy [8]. Cell surface markers, CD133 and CD44, are commonly used to identify CSCs. Cells expressing CD133 and CD44 markers have been shown to possess several characteristics that promote tumor growth and progression, including enhanced spheroid-forming ability and chemoresistance [9,10]. After 48 h of culture in CLs (0–500 g/mL), we employed Western blotting to evaluate CD133 and CD44 expression in H460 and H292 cells. The results indicated that in both H460 and H292 cells, CLs caused a dose-dependent reduction in the expression levels of both markers (Figure 5A,B). Moreover, CL treatment significantly decreased the expression level of the stem cell transcription factor Sox2 compared to the untreated control in both H460 and H292 cells (Figure 5A,B).

The inhibitory effects of CLs on CD133, CD44, and Sox2 were confirmed through immunofluorescence staining experiments. The results showed that CL treatment significantly decreased the levels of Sox2, CD133, and CD44 in H460 cells (Figure 5C). These findings were consistent with the results obtained from the immunofluorescence analysis of H292 cells (Figure 5D). The downregulation of these stemness markers suggests that CL treatment could potentially inhibit the self-renewal and tumorigenicity of lung cancer stem-like cells. These results imply that CLs might have therapeutic potential as anti-lung cancer agents.

### 3.5. CLs Suppress Metastasis and Stemness by Inhibiting the Activation of Akt/mTOR/c-Myc Signaling in NSCLC Cells

**Figure 6:**
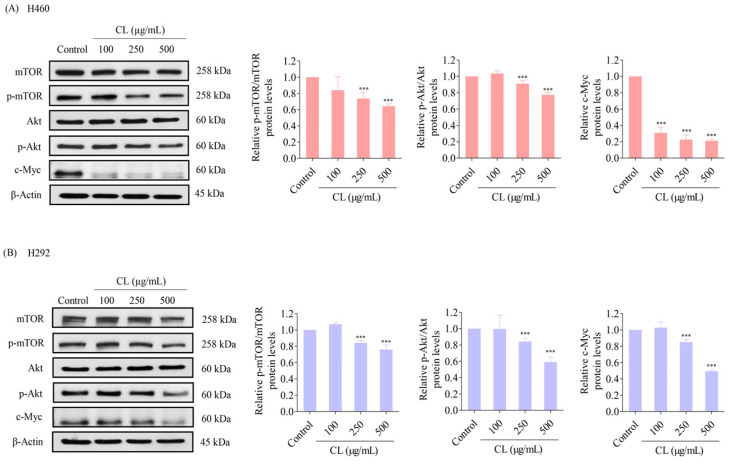
Cholesterol-free shrimp lipids (CLs) inhibit the Akt/mTOR/c-Myc pathway in non-small cell lung cancer (NSCLC) cells. (**A**) H460 and (**B**) H292 cells were treated with various concentrations of CLs (0–500 µg/mL) for 48 h. The expressions of mTOR, p-mTOR, Akt, p-Akt, and c-Myc were assessed by Western blotting. β-actin was utilized as a loading control to ensure equal loading of the protein samples. Densitometry analysis was performed for each protein, and the results are presented as relative protein levels compared to untreated control cells. Uncropped blots can be found in Supplemental Figure S3. Data are represented as the means ± SD (*n* = 3). *** *p* < 0.001 compared with untreated cells.

The upstream regulatory signals of the EMT and CSC phenotypes in NSCLC cells, such as Akt, mTOR, and c-Myc, were further analyzed. H460 and H292 cells were treated with various non-toxic concentrations of CLs (0–500 μg/mL) for 48 h. The protein levels of CSC upstream pathways including Akt, p-Akt, mTOR, and p-mTOR, and cell proliferation protein c-Myc were determined. The results showed that CLs at 100 µg/mL had no effect on p-mTOR/mTOR and p-Akt/Akt protein expression levels in both H460 and H292 cells (Figure 6A,B). However, a significant downregulation of p-mTOR/mTOR and p-Akt/Akt protein expression levels was observed at 250 and 500 µg/mL of CL treatment in the NSCLC cells (H460 and H292) compared with the untreated control (Figure 6A,B). Interestingly, the levels of expression of c-Myc were dramatically reduced after treatment with CLs in both cell lines (Figure 6A,B). These findings suggest that CLs may suppress the EMT and cancer stem cells in NSCLC cells by inhibiting Akt/mTOR signaling pathways and downregulating c-Myc expression.

**Figure 7:**
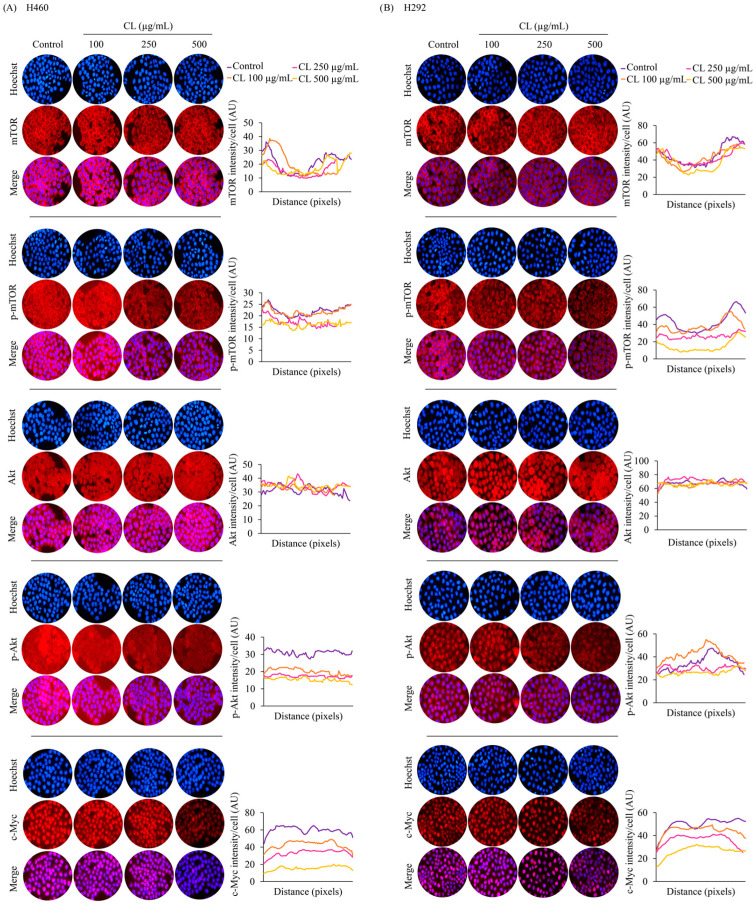
The inhibitory effect of cholesterol-free shrimp lipids (CLs) on the Akt/mTOR/c-Myc pathway in H460 and H292 non-small cell lung cancer (NSCLC) cells. (**A**) H460 and (**B**) H292 cells were treated with various concentrations of CLs (0–500 µg/mL) for 48 h. Fluorescence imaging was conducted to visualize the cellular distributions of mTOR, p-mTOR, Akt, p-Akt, and c-Myc. The red signal represents the staining of corresponding proteins, while the blue signal indicates nuclear DNA staining using Hoechst 33342. The graph illustrates the quantitative analysis of fluorescence intensity for mTOR, p-mTOR, Akt, p-Akt, and c-Myc in H460 and H292 cells. The data were captured using a fluorescence microscope and analyzed with Image J software version 1.52a. Data are represented as the means ± SD (*n* = 3).

Next, we performed immunofluorescence analysis to confirm the levels of essential protein markers, including Akt, p-Akt, mTOR, p-mTOR, and c-Myc. The results confirmed that the protein expression of c-Myc was highly suppressed by CL treatment (Figure 7A,B). These data reveal that CLs are involved in regulating the Akt/mTOR signaling pathway, thereby inhibiting EMT progression and suppressing CSC phenotypes. Furthermore, these results suggest that CLs might suppress the Akt/mTOR signaling pathway and downstream transcription factor c-Myc in NSCLC cells. This suppression could contribute to inhibiting metastasis and stemness-related properties observed in the above experiments.

**Figure 8:**
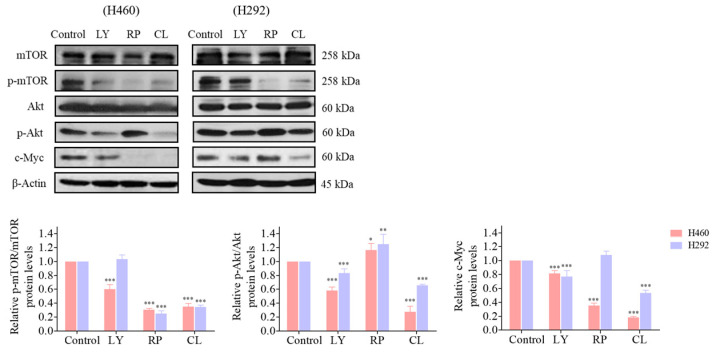
The regulatory effect of cholesterol-free shrimp lipids (CLs) on c-Myc expression via the inhibition of p-Akt and mTOR in H460 and H292 cells. The cells were seeded and treated with LY294002 (5 µM), rapamycin (2 µM), and CL (500 µg/mL) for 48 h. The expressions of mTOR, p-mTOR, Akt, p-Akt, and c-Myc were assessed by Western blotting. β-actin was utilized as a loading control to ensure equal loading of the protein samples. Densitometry analysis was performed for each protein, and the results are presented as relative protein levels compared to untreated control cells. Uncropped blots can be found in Supplemental Figure S4. Data are represented as the means ± SD (*n* = 3). * *p* < 0.05, ** *p* < 0.01, and *** *p* < 0.001 compared with untreated cells.

Based on our findings indicating direct targeting of the Akt protein by CLs (as depicted in Figure 8), the subsequent inactivation of downstream signaling pathways, including a decrease in mTOR, was observed. This highlights the critical role of the Akt/mTOR signaling pathways in controlling c-Myc expression. To further investigate the involvement of Akt in regulating downstream c-Myc expression and its regulation by CL treatment targeting Akt, we conducted Western blotting experiments using LY294002 (2-4-morpholinyl-8-phenlchromone), a PI3K/Akt pathway inhibitor, and rapamycin, an mTOR inhibitor. Treatment of both H460 and H292 cells with LY294002 (5 µM) resulted in a reduction in protein levels of p-Akt, p-mTOR, and c-Myc, confirming the regulatory role of the PI3K/Akt pathway in c-Myc expression. Subsequent treatment with rapamycin (2 µM) led to a significant decrease in p-mTOR levels in both cell lines, corroborating mTOR inhibition. Notably, rapamycin treatment induced an increase in p-Akt levels in both H460 and H292 cells, indicating potential compensatory mechanisms or feedback loop activation upon mTOR inhibition [42,43]. Furthermore, while rapamycin treatment significantly decreased c-Myc levels in H460 cells, the difference in c-Myc expression in H292 cells was not statistically significant, suggesting potential variability in the response to mTOR inhibition among NSCLC cell lines. These findings underscore the significance of regulatory mechanisms governing c-Myc expression mediated by Akt/mTOR signaling pathways.

### 3.6. CLs Suppress CSC Spheroid Formation in NSCLC Cells

**Figure 9:**
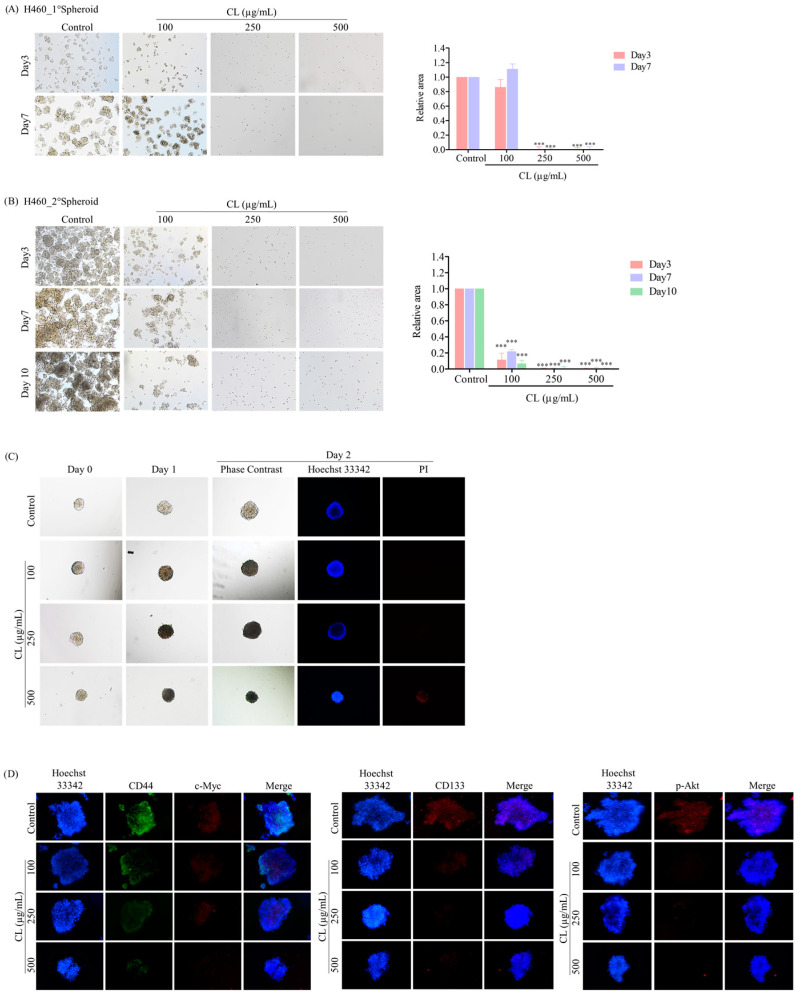
Cholesterol-free shrimp lipids (CLs) suppress cancer stem cell (CSC)-like phenotypes and induce CSC-rich population apoptosis in H460 cells. (**A**) H460 cells were pre-treated with CLs (0–500 µg/mL) and then allowed to generate primary spheroids for 7 days. Primary tumor spheroids were captured after incubation for 3 and 7 days by a phase-contrast microscope. (**B**) The primary tumor spheroids were seeded and incubated for 10 days to generate secondary CSC-enriched spheroids, with images captured on days 3, 7, and 10. The relative areas of primary and secondary spheroids were measured. (**C**) Single CSC-rich secondary spheroids were exposed to CLs (0–500 µg/mL) for 1 and 2 days. Phase-contrast images of secondary spheroids at days 0, 3, and 7 and untreated cells were determined. At 2 days, a single spheroid was stained with Hoechst 33342/propidium iodide (PI). The single spheroid from the CSC-rich population of H460 cells was treated with CLs (0–500 µg/mL) for 24 h. (**D**) Immunofluorescence staining was performed to assess the levels and distributions of CD44, c-Myc, CD133, and p-Akt. The nuclei of cells were stained with Hoechst33342, while CD44 was specifically labeled with an Alexa Fluor 488-labeled secondary antibody. Additionally, c-Myc, CD133, and p-Akt were stained using the Alexa Fluor 594-labeled secondary antibody. The data represent the means ± SD (*n* = 3). *** *p* < 0.001, compared to the untreated cells.

**Figure 10:**
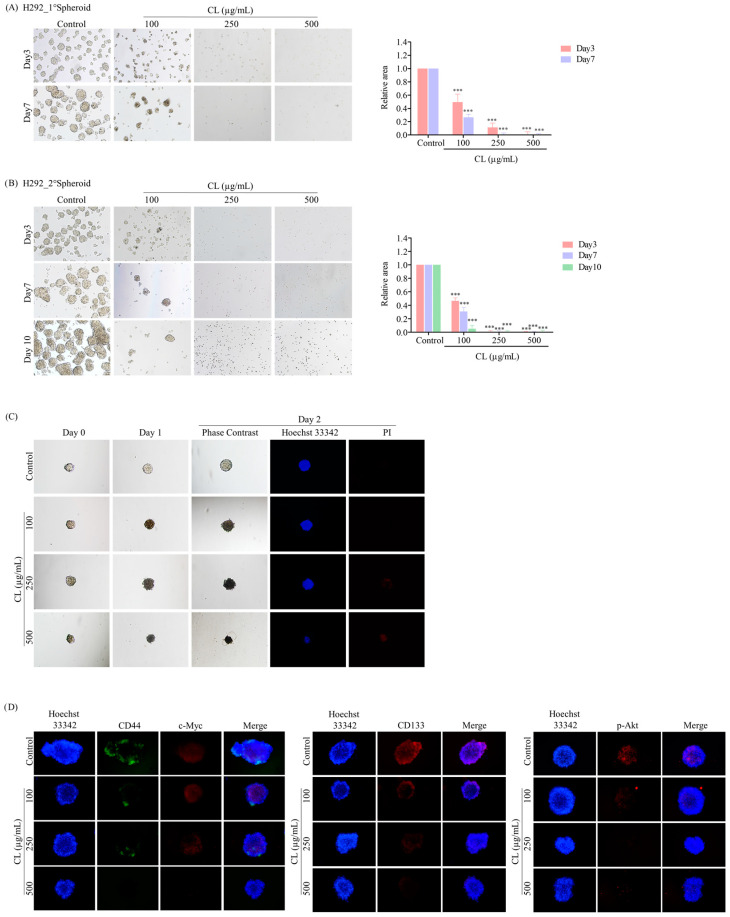
Cholesterol-free shrimp lipids (CLs) suppress cancer stem cell (CSC)-like phenotypes and induce CSC-rich population apoptosis in H292 cells. (**A**) H292 cells were pre-treated with CLs (0–500 µg/mL) and then allowed to generate primary spheroids for 7 days. Primary tumor spheroids were captured after incubation for 3 and 7 days by a phase-contrast microscope. (**B**) The primary tumor spheroids were seeded and incubated for 10 days to generate secondary CSC-enriched spheroids, with images captured on days 3, 7, and 10. The relative areas of primary and secondary spheroids were measured. (**C**) Single CSC-rich secondary spheroids were exposed to CLs (0–500 µg/mL) for 1 and 2 days. Phase-contrast images of secondary spheroids at days 0, 3, and 7 and untreated cells were determined. At 2 days, a single spheroid was stained with Hoechst 33342/propidium iodide (PI). The single spheroid from the CSC-rich population of H292 cells was treated with CLs (0–500 µg/mL) for 24 h. (**D**) Immunofluorescence staining was performed to assess the levels and distribution of CD44, c-Myc, CD133, and p-Akt. The nuclei of cells were stained with Hoechst33342, while CD44 was specifically labeled with an Alexa Fluor 488-labeled secondary antibody. Additionally, c-Myc, CD133, and p-Akt were stained using the Alexa Fluor 594-labeled secondary antibody. The data represent the means ± SD (*n* = 3). *** *p* < 0.001, compared to the untreated cells.

After investigating a two-dimensional (2D) cell culture model, we assessed the ability of CLs to inhibit CSC-like phenotypes in NSCLC by employing a three-dimensional (3D) tumor spheroid formation assay. First, H460 and H292 lung cancer cells were pre-treated with non-cytotoxic concentrations of CLs ranging from 0 to 500 µg/mL for 48 h. Subsequently, the cells were cultured at a low density and allowed to form first-generation spheres for seven days. The formation of secondary spheroids from single cells derived from primary spheroids was allowed for 10 days. The results showed that the control cells had a high ability to form tumor spheroids, while cells treated with CLs exhibited a decreased ability to form these spheroids (Figure 9A and Figure 10A). This suggests that CLs had a suppressive effect on the CSC populations in these cells. Specifically, at concentrations of 250 and 500 µg/mL, which were considered non-cytotoxic, CLs significantly decreased the number and size of primary and secondary spheroids compared to the control in both lung cancer H460 (Figure 9A,B) and H292 cells (Figure 10A,B).

After 10 days, the secondary spheroids H460 and H292, which had comparable dimensions and shapes, were chosen. These spheroids were then treated with CLs at varying dosages (ranging from 0 to 500 g/mL), and their responses were monitored on days 0, 1, and 2. Individual CSC spheroids were visible in both the control and CL-treated cells on days 0, 1, and 2 (Figure 9C and Figure 10C). At the highest dose, CL treatment of CSC spheres drastically decreased the CSC populations in both H460 and H292 cells and resulted in a significant reduction in the size of CSC spheres in both cell lines, as shown in Figure 9C and Figure 10C, respectively. Interestingly, we observed that the spheroids were stained with PI after CL treatment at 250 and 500 µg/mL in both H460 (Figure 9C) and H292 (Figure 10C), indicating that CL exposure induced the death of CSC spheres.

Furthermore, CLs were demonstrated to reduce the expression of CSC markers, including CD44 and CD133, and strongly inhibit c-Myc and p-Akt expression in both H460 (Figure 9D) and H292 (Figure 10D) CSC spheres. These results indicate that CLs have the ability to suppress the CSC-like properties of NSCLC cells in 3D culture conditions.

## 4. Discussion

Shrimp heads, well known as a byproduct of the shrimp processing industry, contain high levels of carotenoids, including astaxanthin and astaxanthin esters. These compounds are powerful antioxidants that have been linked to several health benefits, such as reducing inflammation, improving immune function, and suppressing the development of different types of cancer [22,23,24]. Additionally, shrimp head oil is rich in PUFAs, particularly EPA and DHA, which are essential omega-3 fatty acids crucial for maintaining heart health and brain function and reducing inflammation [25]. PUFA uptake has also been inversely related to lung, prostate, breast, and colorectal cancers [44,45]. In lung cancer, several studies have shown the potential effects of omega-3 and omega-6 PUFAs in suppressing proliferation, promoting apoptosis, and inhibiting angiogenesis [46,47]. Moreover, n-3 PUFAs enhanced the sensitivity of breast cancer, sarcoma, and leukemia in in vivo models of chemotherapeutic agents [28]. However, the consumption of shrimp oil and lipids has been restricted due to the presence of cholesterol. The American Heart Association recommends that individuals consume no more than 300 mg of cholesterol per day [48]. Nevertheless, individuals with high blood cholesterol levels, diabetes, or a history of cardiovascular disease may need to further reduce their intake to no more than 200 mg/day.

Migration and invasion of cancer cells are major factors that contribute to the poor prognosis of many types of cancer [49]. Targeting the process of EMT, which is associated with the migration and invasion of cancer cells, is an important strategy for developing new cancer therapies to treat metastasis. In lung cancer, EMT plays a critical role in cancer progression and metastasis [7]. By inhibiting this process, it may be possible to prevent cancer cells from acquiring the migratory and invasive properties needed for metastasis. There are several potential targets for inhibiting EMT in lung cancer, including various signaling pathways and transcription factors that regulate EMT-related gene expression [8,9]. For example, the TGF beta signaling pathway has been implicated in EMT induction and cancer metastasis [50]. Inhibitors targeting this pathway are currently under development as potential cancer therapeutics. Additionally, several transcription factors, such as Snail, Slug, and Twist, are known to regulate EMT gene expression and are potential targets for cancer therapy [8]. In this study, CL treatment inhibited the migratory activity of human NSCLC (Figure 3A–C). From a mechanistic approach, CLs were shown to inhibit EMT in these cells, as indicated by the depletion of Vimentin, a hallmark of mesenchymal cells, and the deregulation of the levels of the EMT transcription factors Snail and Slug in both H460 and H292 cells (Figure 4A–D). These results are consistent with previous studies showing that astaxanthin and PUFAs, which are dominant compounds derived from shrimp lipids, suppress cancer cell migration [51,52].

CD133 and CD44 are cell surface markers commonly used to identify CSCs [9,10]. CD133, also known as Prominin-1, is a transmembrane glycoprotein that plays a crucial role in tumor recurrence, metastasis, and resistance to chemotherapy and radiation therapy [53]. CD44, on the other hand, is a transmembrane glycoprotein that is involved in cell–cell interactions, cell adhesion, and cell migration [54]. These biomarkers have been shown to be overexpressed on the surfaces of CSCs in several types of cancer, including breast, pancreatic, and lung cancers [53,54]. Studies have shown that the upregulation of CD133 and CD44 in lung cancer cells is associated with the induction of EMT, which is a critical step in the development of metastasis [9,10,11]. The suppression of CD133 and CD44 expression can decrease the formation of tumors and spheres, which are indicative of the self-renewal capacity of CSCs. In lung cancer cells, the downregulation of CD133 and CD44 expression has been shown to reduce the number of tumor spheres formed in vitro and to decrease tumor growth in vivo [10,55]. Sox2 is a transcription factor that plays a critical role in the self-renewal and maintenance of stem cells, including CSCs [12]. Sox2 has also been identified as a key regulator of the stemness and tumorigenic potential of CSCs in several forms of tumors [56]. Inhibition of Sox2 has demonstrated promising results in targeting lung cancer CSCs [57]. A recent report by Chen et al. showed that the knockdown of Sox2 expression using small interfering RNA (siRNA) in lung cancer CSCs led to a reduction in tumor sphere formation and cell proliferation [58]. Additionally, it increased sensitivity to cisplatin, a commonly used chemotherapy drug. These findings suggest that targeting CD133, CD44, and Sox2 expression or downstream signaling pathways might be promising strategies for developing new cancer therapies that target lung cancer CSCs. In the current study, we found that the expression of stemness markers CD133, CD44, and Sox2 was downregulated in response to CL treatment in both H460 and H292 cells (Figure 5A–D). Additionally, the CL treatment suppressed spheroid formation and induced lung CSC death by decreasing the expression of CD44 and CD133 (Figure 8 and Figure 9). To support the effect of CLs in inhibiting CSC, it was reported that astaxanthin has the potential to reduce the populations of BT20 and T47D breast CSCs and suppress stemness markers [59]. Similarly, DHA and EPA were found to suppress the development of cancer stem-like cells in colorectal cancer by inducing apoptosis [60].

Activated Akt is essential for the EMT process, which is a critical step in cancer metastasis [61]. Akt has been shown to regulate EMT by controlling the expression of various EMT-related transcription factors, such as Snail, Slug, and Twist [17]. In addition, Akt signaling has been shown to regulate CSC self-renewal and maintenance in various types of cancers, including lung cancer [14]. Akt inhibition has been shown to decrease the expression of stem cell markers such as CD133, CD44, and ALDH and to reduce the proportion of CSCs in lung cancer cell lines and patient-derived xenografts [10,62]. Studies have shown that inhibiting Akt signaling can lead to the downregulation of Sox2 expression and the depletion of lung CSCs in NSCLC [16]. Similarly, the suppression of mTOR signaling has been found to reverse EMT and decrease the CSC phenotype in lung cancer cells, as mTOR is a downstream target of Akt [19]. The Akt/mTOR signaling pathway can activate c-Myc, which plays a critical role in promoting the self-renewal and survival of CSCs in various cancers, including lung cancer [63]. c-Myc has been shown to regulate the expression of pluripotent transcription factors, such as Oct4, Sox2, and Nanog, and to promote the proliferation and survival of CSCs [16]. In addition, c-Myc can induce angiogenesis, promoting tumor growth and metastasis. In agreement with these observations, this study showed that CLs decreased the amounts of active Akt, mTOR, and c-Myc (Figure 6 and Figure 7), subsequently reducing EMT markers Vimentin, Snail, and Slug (Figure 4) and inhibiting CSC phenotype acquisition (Figure 8 and Figure 9). This suggests that the Akt/mTOR/c-Myc pathway and its downstream targets, including EMT and CSC markers, were regulated by the CLs. In addition, this comparison revealed that CL treatment produced comparable outcomes to earlier research on cancer development (Table 2). These effects parallel the findings observed with astaxanthin and PUFAs, suggesting potential similarities in their mechanisms of action.

Consistent with our observations, numerous other studies have investigated the effects of PI3K/Akt and mTOR inhibitors on cancer progression [42,64], further supporting the significance of Akt and mTOR inhibition in mediating the observed effects of CLs. LY294002, a specific inhibitor of the PI3K/Akt pathway, has been extensively studied in various cancer models, where it has demonstrated its efficacy in suppressing Akt signaling and downstream oncogenic processes [64]. LY294002 has demonstrated its efficacy in inhibiting cell proliferation, inducing apoptosis, and suppressing tumor growth, while also attenuating EMT by suppressing the Akt-mediated regulation of EMT-related transcription factors and signaling pathways [65]. Similarly, rapamycin has exhibited potent anticancer effects through the inhibition of mTOR signaling, leading to reduced tumor growth and metastasis [42,43]. In addition, both LY294002 and rapamycin have been implicated in modulating c-Myc expression in various cancer types [64,66].

## 5. Conclusions

**Figure 11:**
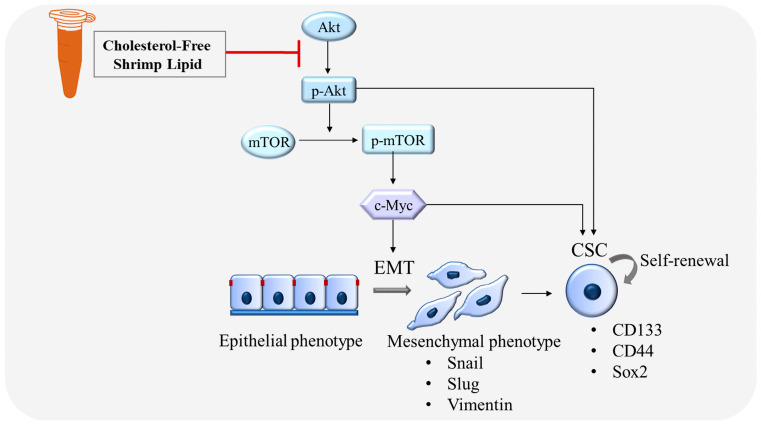
Schematic mechanism of CLs suppressing EMT and CSC in lung cancer cells.

In conclusion, this study highlighted the potential health benefits of lipids derived from shrimp, particularly CLs, in suppressing key factors associated with cancer progression and metastasis. This investigation has, for the first time, revealed the potential of CLs to inhibit the migration of human lung cancer cells (H460 and H292) by targeting EMT. Furthermore, CLs exhibit promising effects on CSCs, as evidenced by the downregulation of stemness markers CD133, CD44, and Sox2 and the inhibition of CSC-induced spheroid formation. The underlying mechanism of action of CLs has been postulated to involve its capacity to inhibit Akt activation. Consequently, this leads to a reduction in the active phosphorylated Akt as well as its downstream targets, such as mTOR and c-Myc (as illustrated in Figure 11). The suppression of the Akt/mTOR/c-Myc pathway confers a pharmacological effect of CLs on impeding EMT and CSC, which are drivers of cancer metastasis. Nevertheless, the translation of these promising results into clinical applications requires further validation through comprehensive investigations and clinical trials. This study emphasizes the significance of exploring shrimp-derived lipids as a possible source of innovative anticancer compounds, providing new opportunities for advancements in cancer treatment.

## Figures and Tables

**Table 1:** Fatty acid compositions of cholesterol free shrimp lipids (CLs).

Fatty Acid	Amount (g/100 g Lipid)
C14:0	1.41 ± 0.02
C15:0	0.75 ± 0.01
C16:0	15.30 ± 0.14
C16:1	1.36 ± 0.01
C17:0	1.67 ± 0.02
C17:1	0.54 ± 0.03
C18:0	10.59 ± 0.09
C18:1	12.28 ± 0.12
C18:2	13.77 ± 0.11
C20:0	1.16 ± 0.01
C20:1	1.39 ± 0.00
C18:3	0.98 ± 0.04
C20:2	2.74 ± 0.02
C23:0	6.14 ± 0.03
C24:0	1.11 ± 0.02
C20:5	7.78 ± 0.01
C24:1	1.13 ± 0.02
C22:6	8.37 ± 0.15

Values are expressed as means ± standard deviations (*n* = 3).

**Table 2:** Comparison of the effects of CL treatment with previous studies on cancer progression.

Effect of CL Treatment	Comparison Studies	References
Inhibition of NSCLC Migration	- Astaxanthin suppressed breast cancer cell proliferation and migration. - DHA and EPA inhibited non-small cell lung cancer cell proliferation and progression via the PI3K/Akt pathway.	[51] [52]
Inhibition of EMT Markers	- Astaxanthin and PUFAs induced the downregulation of EMT markers.	[51,52]
Inhibition of CSC Phenotype	- Astaxanthin reduced the populations of BT20 and T47D breast cancer stem cells and suppressed stemness markers. - DHA and EPA suppressed cancer stem-like cells in colorectal cancer.	[59] [60]

## Data Availability

The data mentioned in this work are available from the corresponding author upon reasonable request.

## Supplementary Materials

The following supporting information can be downloaded at: https://www.mdpi.com/article/10.3390/biomedicines12040722/s1, Figure S1: The uncropped blotting bands of Figure 4; Figure S2: The uncropped blotting bands of Figure 5; Figure S3: The uncropped blotting bands of Figure 6; Figure S4: The uncropped blotting bands of Figure 8.
