# Correction: Thepthanee et al. Shrimp Lipids Inhibit Migration, Epithelial–Mesenchymal Transition, and Cancer Stem Cells via Akt/mTOR/c-Myc Pathway Suppression. *Biomedicines* 2024, *12*, 722

**DOI:** 10.3390/biomedicines12081752

**Published:** 2024-08-05

**Authors:** Chorpaka Thepthanee, Zin Zin Ei, Soottawat Benjakul, Hongbin Zou, Korrakod Petsri, Bhurichaya Innets, Pithi Chanvorachote

**Affiliations:** 1 Department of Food Science, School of Food Industry, King Mongkut’s Institute of Technology Ladkrabang, Bangkok 10520, Thailand; chorpaka.thep@gmail.com; 2 Center of Excellence in Cancer Cell and Molecular Biology, Faculty of Pharmaceutical Sciences, Chulalongkorn University, Bangkok 10330, Thailand; hushushin@gmail.com (Z.Z.E.); 6481004120@student.chula.ac.th (B.I.); 3 Department of Pharmacology and Physiology, Faculty of Pharmaceutical Sciences, Chulalongkorn University, Bangkok 10330, Thailand; 4 International Center of Excellence in Seafood Science and Innovation, Faculty of Agro-Industry, Prince of Songkhla University, Songkhla 90110, Thailand; soottawat.b@psu.ac.th; 5 College of Pharmaceutical Sciences, Zhejiang University, Hangzhou 310058, China; zouhb@zju.edu.cn; 6 Department of Pharmacology, Faculty of Medicine, Kasetsart University, Bangkok 10900, Thailand; korrakod.petsri@gmail.com

**Error in Figure**

In the original publication [1], there was a mistake in Figure 4 as published. Specifically, there was an issue with duplicated subfigures in Figure 4C. Additionally, the β-actin bands in Figures 4 and 5 appeared similar, which may have resulted from an error during figure preparation. The corrected Figure 4 appears below. The authors state that the scientific conclusions are unaffected. This correction was approved by the Academic Editor. The original publication has also been updated.

## Figures and Tables

**Figure 4:**
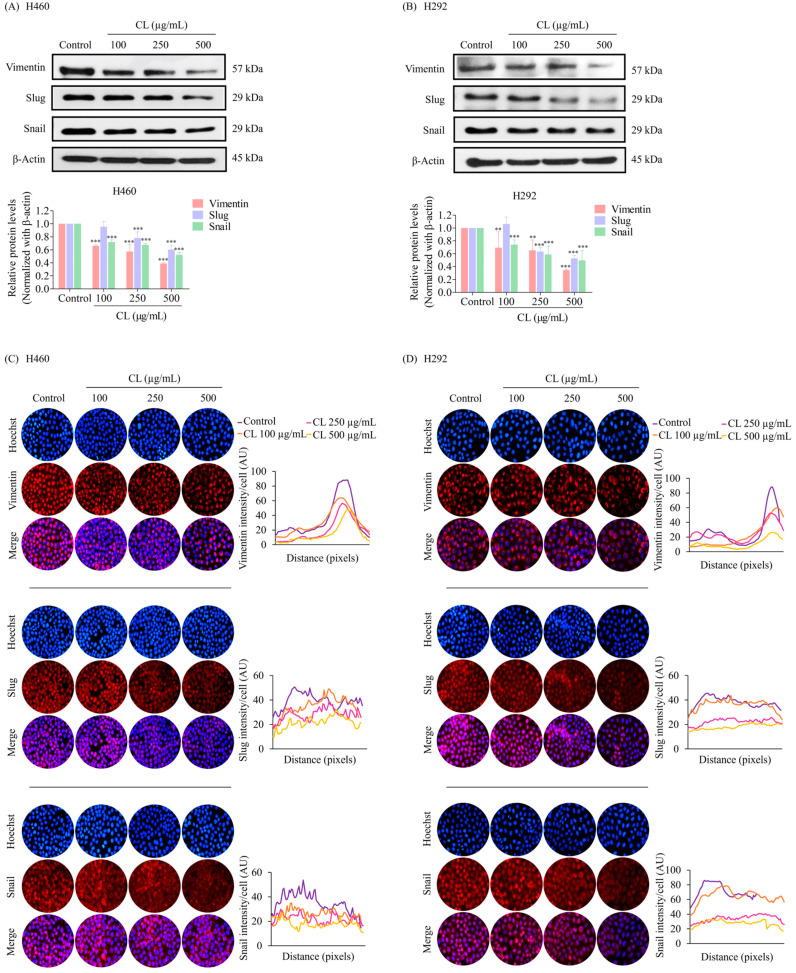
Cholesterol-free shrimp lipids (CLs) reduce the expression of the epithelial–mesenchymal transition (EMT) marker in non-small cell lung cancer (NSCLC) cells. (**A**) H460 and (**B**) H292 cells were treated with various concentrations of CLs (0–500 µg/mL) for 48 h. The expressions of key EMT markers, including Vimentin, Slug, and Snail, were assessed by Western blotting. β-actin was utilized as a loading control to ensure equal loading of the protein samples. Densitometry analysis was performed for each protein, and the results are presented as relative protein levels compared to untreated control cells. Uncropped blots can be found in Supplemental Figure S1. Immunofluorescence confirmation of decreased the expression of EMT markers (Vimentin, Slug, and Snail) in (**C**) H460 and (**D**) H292 cells treated with CLs. This analysis included the localization and expression patterns of Vimentin, Slug, and Snail. H460 and H292 cells were treated with various concentrations of CLs (0–500 µg/mL) for 48 h. Fluorescence imaging was conducted to visualize the cellular distributions of Vimentin, Slug, and Snail. The red signal represents the staining of corresponding proteins, while the blue signal indicates nuclear DNA staining using Hoechst 33342. The graph illustrates the quantitative analysis of the fluorescence intensities of Vimentin, Slug, and Snail in H460 and H292 cells. The data were captured using a fluorescence microscope and analyzed with Image J software version 1.52a. Data are represented as the means ± SD (*n* = 3). ** *p* < 0.01 and *** *p* < 0.001 compared with untreated cells.
