# Supplementary material for: Shrimp Lipids Inhibit Migration, Epithelial–Mesenchymal Transition, and Cancer Stem Cells via Akt/mTOR/c-Myc Pathway Suppression
Source: Biomedicines. 2024 Mar 25;12(4):722. doi: 10.3390/biomedicines12040722 (PMC11048134; doi:10.3390/biomedicines12040722)
Supplement: Supplementary file 1 [file biomedicines-12-00722-s001.zip › biomedicines-2889643-supplementary.pdf]

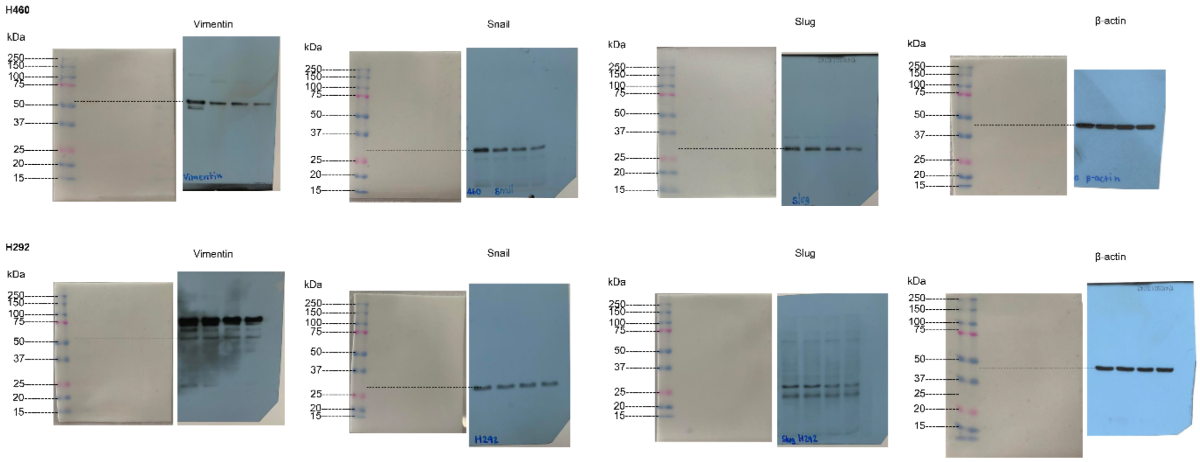

Figure S1. The uncropped blotting bands of Figure 4.

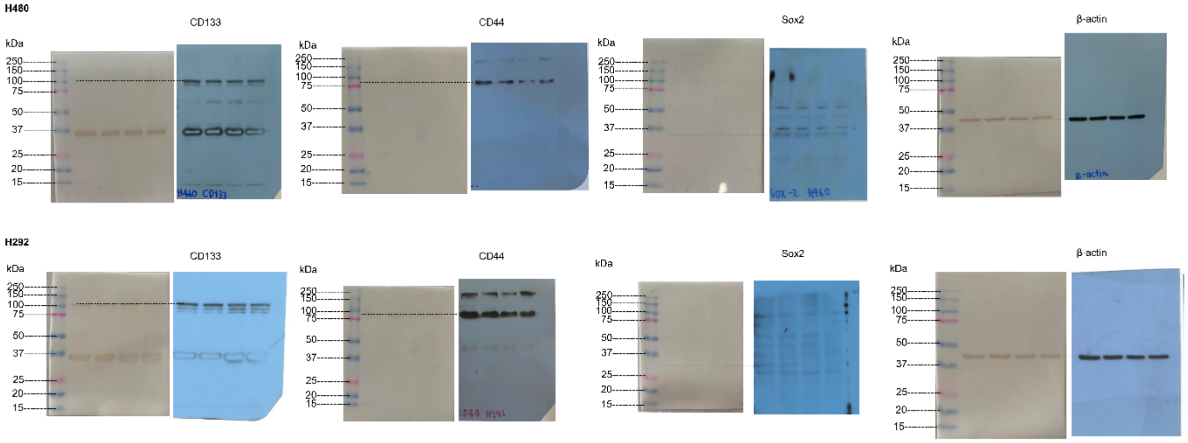

Figure S2. The uncropped blotting bands of Figure 5.

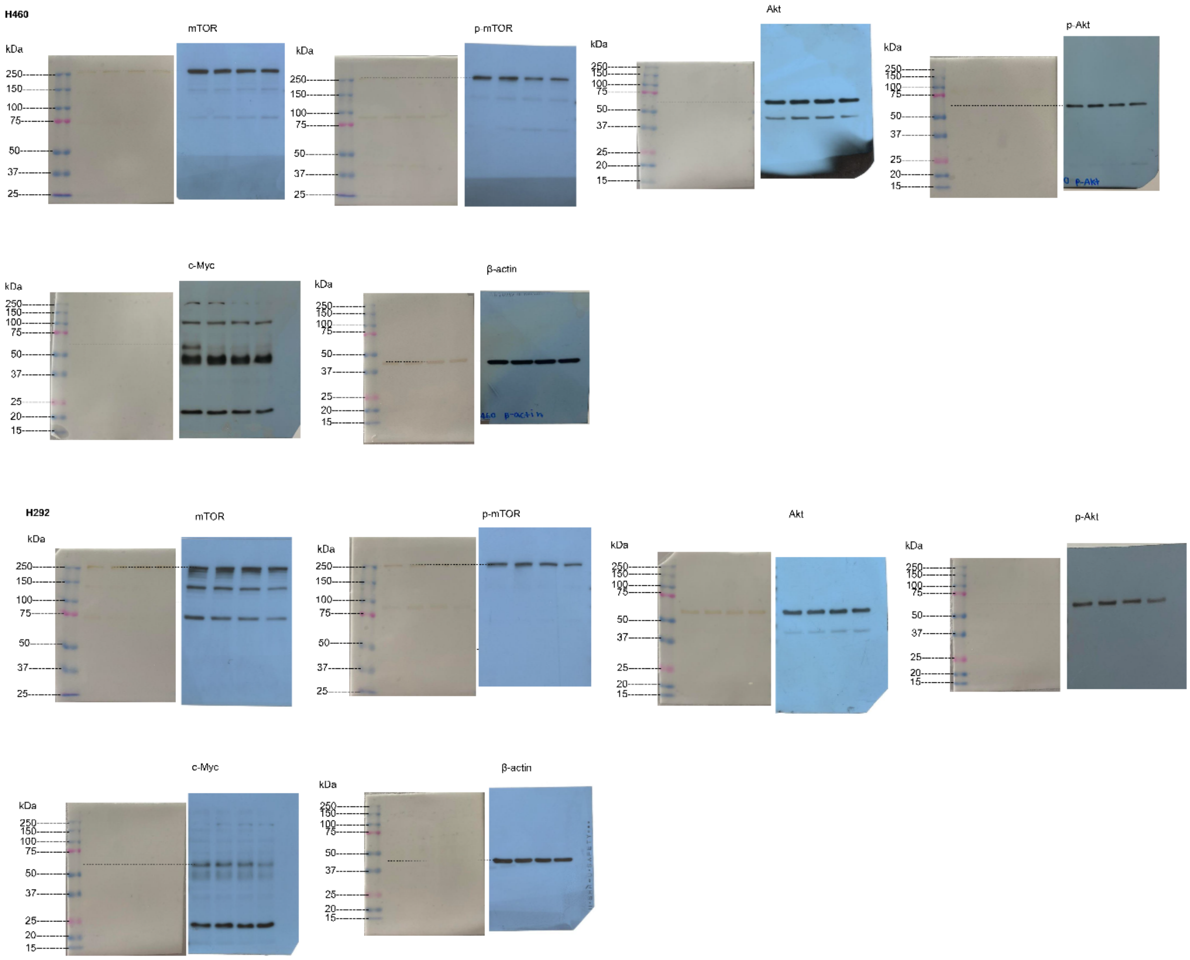

Figure S3. The uncropped blotting bands of Figure 6.

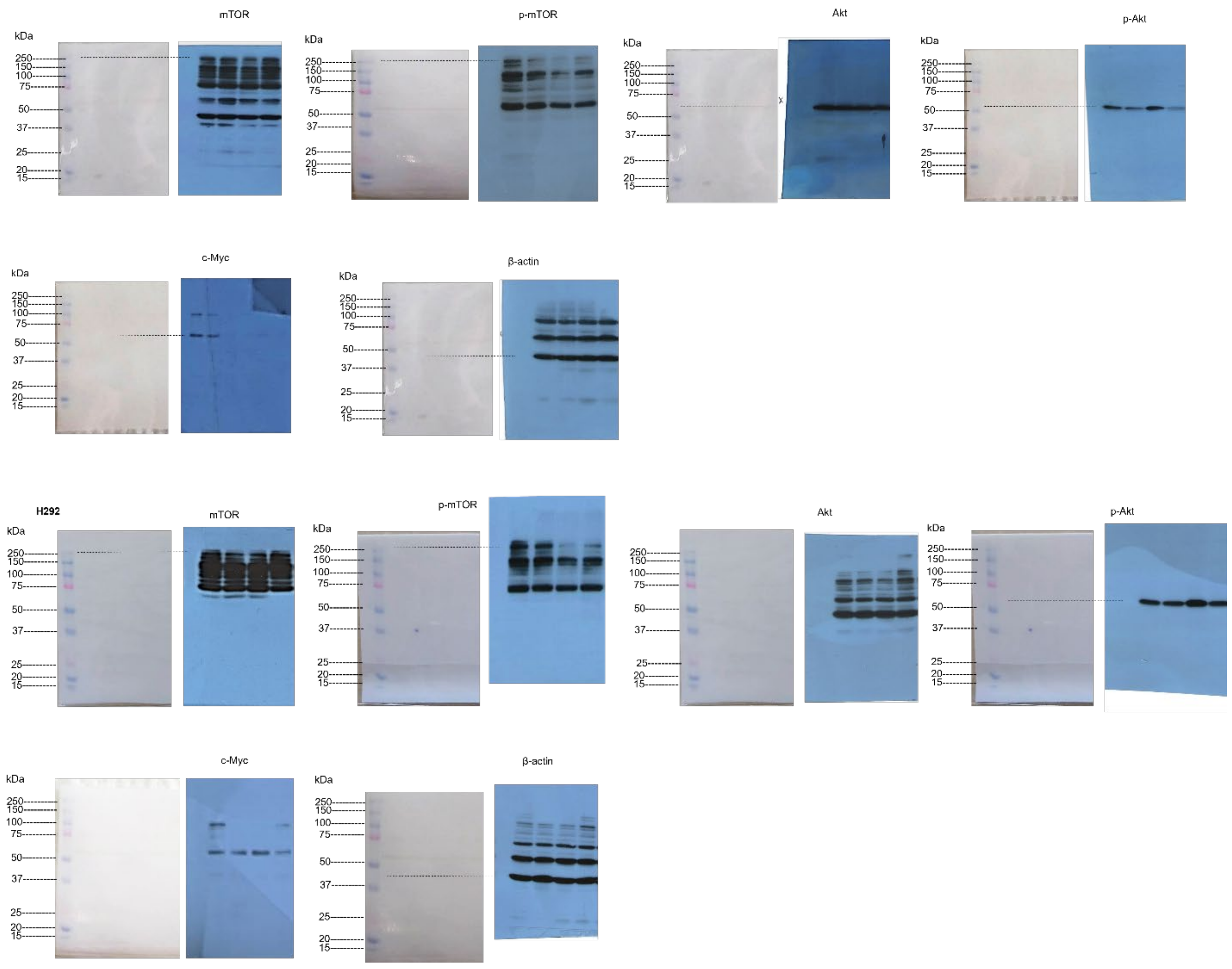

Figure S4. The uncropped blotting bands of Figure 8.
